# Discovery of *Ganoderma lucidum* triterpenoids as potential inhibitors against Dengue virus NS2B-NS3 protease

**DOI:** 10.1038/s41598-019-55723-5

**Published:** 2019-12-13

**Authors:** Shiv Bharadwaj, Kyung Eun Lee, Vivek Dhar Dwivedi, Umesh Yadava, Aleksha Panwar, Stuart. J. Lucas, Amit Pandey, Sang Gu Kang

**Affiliations:** 1 Department of Biotechnology, Institute of Biotechnology, College of Life and Applied Sciences, Yeungnam University, 280 Daehak-Ro, Gyeongsan, Gyeongbuk 38541 Republic of Korea; 2 Centre for Bioinformatics, Computational and Systems Biology, Pathfinder Research and Training Foundation, Greater Noida, India; 3 Department of Physics, Deen Dayal Upadhyay Gorakhpur University, Gorakhpur, India; 4 Clinical and Cellular Virology Lab, Translational Health Science and Technology Institute, NCR-Biotech Science Cluster, Faridabad-Gurgaon Highway, Faridabad, 121001 India; 5 Sabanci University Nanotechnology Research and Application Centre (SUNUM), Istanbul, Turkey; 6 Forest Pathology Division, Forest Research Institute, Dehradun, India

**Keywords:** Protein structure predictions, Applied microbiology

## Abstract

Dengue virus (DENV) infection causes serious health problems in humans for which no drug is currently available. Recently, DENV NS2B-NS3 protease has been proposed as a primary target for anti-dengue drug discovery due to its important role in new virus particle formation by conducting DENV polyprotein cleavage. Triterpenoids from the medicinal fungus *Ganoderma lucidum* have been suggested as pharmacologically bioactive compounds and tested as anti-viral agents against various viral pathogens including human immunodeficiency virus. However, no reports are available concerning the anti-viral activity of triterpenoids from *Ganoderma lucidum* against DENV. Therefore, we employed a virtual screening approach to predict the functional triterpenoids from *Ganoderma lucidum* as potential inhibitors of DENV NS2B-NS3 protease, followed by an *in vitro* assay. From *in silico* analysis of twenty-two triterpenoids of *Ganoderma lucidum*, four triterpenoids, viz. Ganodermanontriol (−6.291 kcal/mol), Lucidumol A (−5.993 kcal/mol), Ganoderic acid C2 (−5.948 kcal/mol) and Ganosporeric acid A (−5.983 kcal/mol) were predicted to be viral protease inhibitors by comparison to reference inhibitor 1,8-Dihydroxy-4,5-dinitroanthraquinone (−5.377 kcal/mol). These results were further studied for binding affinity and stability using the molecular mechanics/generalized Born surface area method and Molecular Dynamics simulations, respectively. Also, *in vitro* viral infection inhibition suggested that Ganodermanontriol is a potent bioactive triterpenoid.

## Introduction

Dengue virus (DENV) belongs to the Flaviviridae family and is a lethal microbe, transmitted by *Aedes albopictus* and *Aedes aegypti* mosquitoes^1–3^, which causes Dengue Hemorrhagic Fever^4,5^ and Dengue Shock Syndrome^6,7^. Both types of dengue fever are potentially deadly infections caused by five different serotypes of DENV^8,9^. DENV contains 10,723 nucleotides on its single stranded positive RNA genome and encodes a 3391-amino acid monomeric polyprotein as a precursor of the virus. The translated DENV polyprotein contains seven non-structural proteins and three structural proteins^8,10^. Each protein performs a specific function towards the generation of new virus particles, which also employs host cell machinery. The NS3 protease (NS3pro) domain, a member of the S7 family of serine proteases that are brought into their fully active form by binding with cofactor NS2B, mediates the processing of the polyprotein at specific sites. Thus, the NS2B-NS3pro enzyme of DENV has been perceived as an ideal target for the development of new anti-DENV drugs^11–13^. The molecular mechanism of dengue virus protease and its inhibitors with medicinal chemistry perspective has been summarized in the review^14^. In this context, natural products have attracted considerable interest as a pool of novel medicinal compounds^15^. For instance, secondary metabolites from several plant fungal pathogens have been approved as medicinal compounds against various diseases and infections^16–19^. Natural compounds have distinct advantages over synthetic chemistry methods for drug discovery, as they may include druglike properties, biocompatibility and novel structures that are difficult to synthesize *in vitro*^20,21^. For instance, inhibition of DENV protease by thioguanine, an analogue of naturally occurring purine base guanine was recently reported^22^. Additionally, computer-aided drug design approaches for drug discovery have gained importance in hit identification and lead optimization against various drug-targetable receptors^23,24^. Thus, computational approaches may be beneficial in the discovery of novel bioactive compounds as NS2B-NS3pro inhibitors.

*Ganoderma lucidum* (*G. lucidum*) is one of the best-known medicinal fungus species and has been employed as a therapeutic agent against various disorders^25–28^. Recently, this fungus has been well recognized for its pharmacological activities, as reflected by its inclusion in the American Herbal Pharmacopoeia and Therapeutic Compendium and for whole genome sequencing^29,30^. Chemical investigations of the fruiting body, mycelia, and spores have revealed that it contains a large reservoir of bioactive compounds; approximately 400 compounds including triterpenes, polysaccharides, sterols, and peptides have been identified^31,32^. However, triterpenoids and polysaccharides were suggested as the most important pharmacologically active compounds in *G. lucidum*^29^. It has been reported that *G. lucidum* compounds have several medicinal properties such as anti-tumor^33^, anti-microbial^28^, anti-atherosclerotic^34^, anti-inflammatory, hypolipidemic^35^, anti-diabetic, anti-oxidative, radical-scavenging and anti-aging activities^33^. Moreover, antiviral activity of *G. lucidum* triterpenoids have been documented against various pathogenic viruses such as herpes simplex virus types 1 (HSV-1 and HSV-2), influenza A virus (Flu A), vesicular stomatitis virus (VSV) and human immunodeficiency virus (HIV)^24,36,37^. However, the antiviral activity of triterpenoids from *G. lucidum* against dengue virus (DENV) has not yet been reported. Moreover, in the absence of any specific drug against DENV infection, triterpenoids from *G. lucidum* could be promising in the development of potential drugs against DENV-induced disorders.

**Figure 1:**
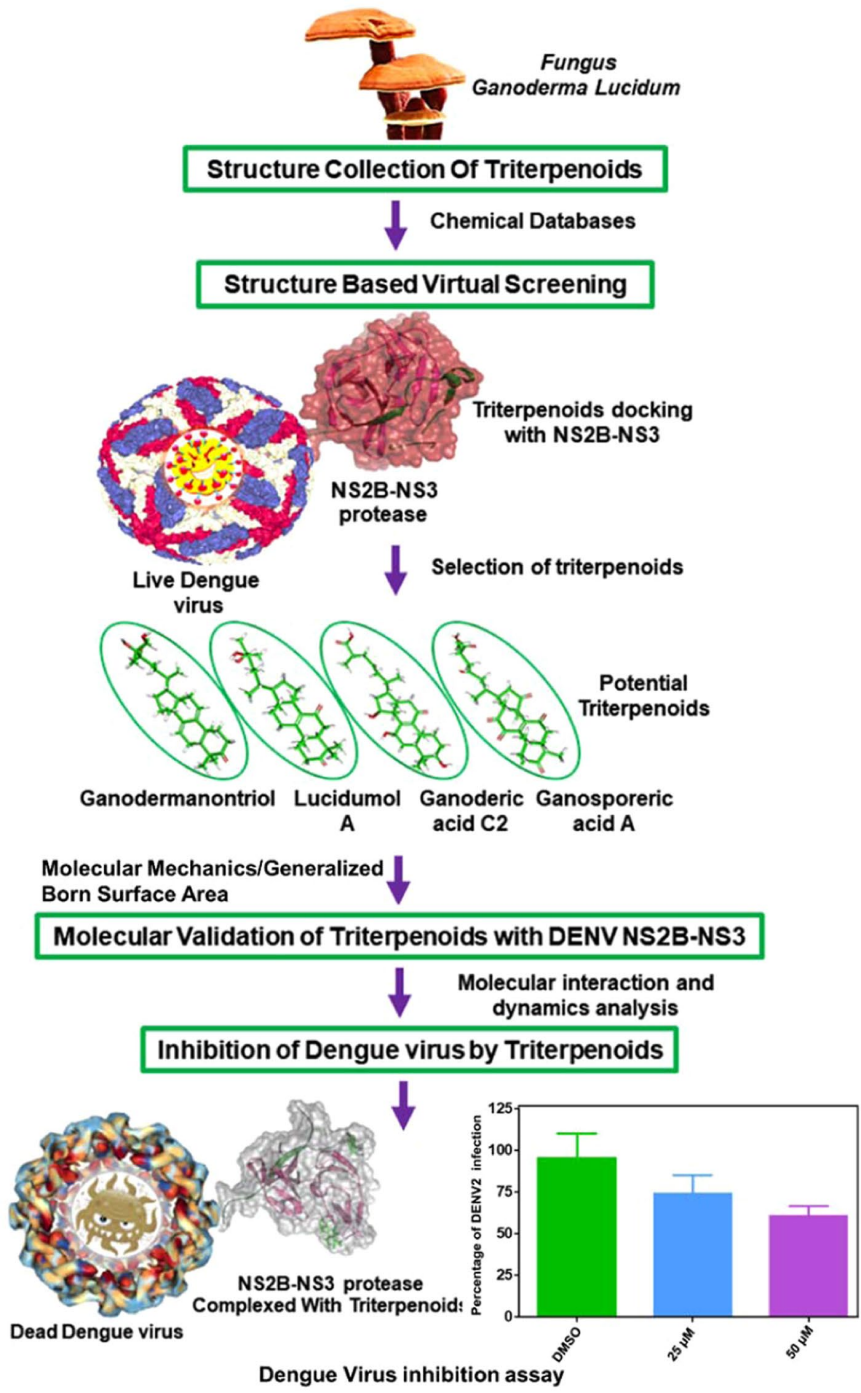
Schematic representation of different steps followed for the discovery of functional triterpenoids from *Ganoderma lucidum* against DENV infection through inhibition of NS2B-NS3 protease.

For a decade, molecular docking approach has been widely used in structure-based drug design due to its ability to calculate the probable accuracy and interaction profile of small molecules as ligands at the active site of the target protein^38^, and further validation by employing molecular dynamics simulation^39^. Considering the important role of NS2B-NS3 protease in DENV infection, identification of bioactive triterpenoids from *G. lucidum* that can inhibit NS2B-NS3 protease activity was proposed as an essential step towards the discovery of DENV inhibitors. Furthermore, to increase the probability of finding triterpenoids from *G. lucidum* that can act as protease inhibitors during dengue infection, we retrieved triterpenoids from the literature that have been used in antiviral studies. Hence, this study includes initial screening of selected triterpenoids against the active site of DENV NS2B-NS3 protease using structure-based screening in the Glide module and validation by molecular dynamics simulation in the Desmond module of the Schrodinger suite. The screened triterpenoids with high potential binding scores were also studied using an *in vitro* assay for DENV inhibition. The various steps of the present study are depicted in Fig. 1.

## Results and Discussion

### NS2B-NS3 protease

**Figure 2:**
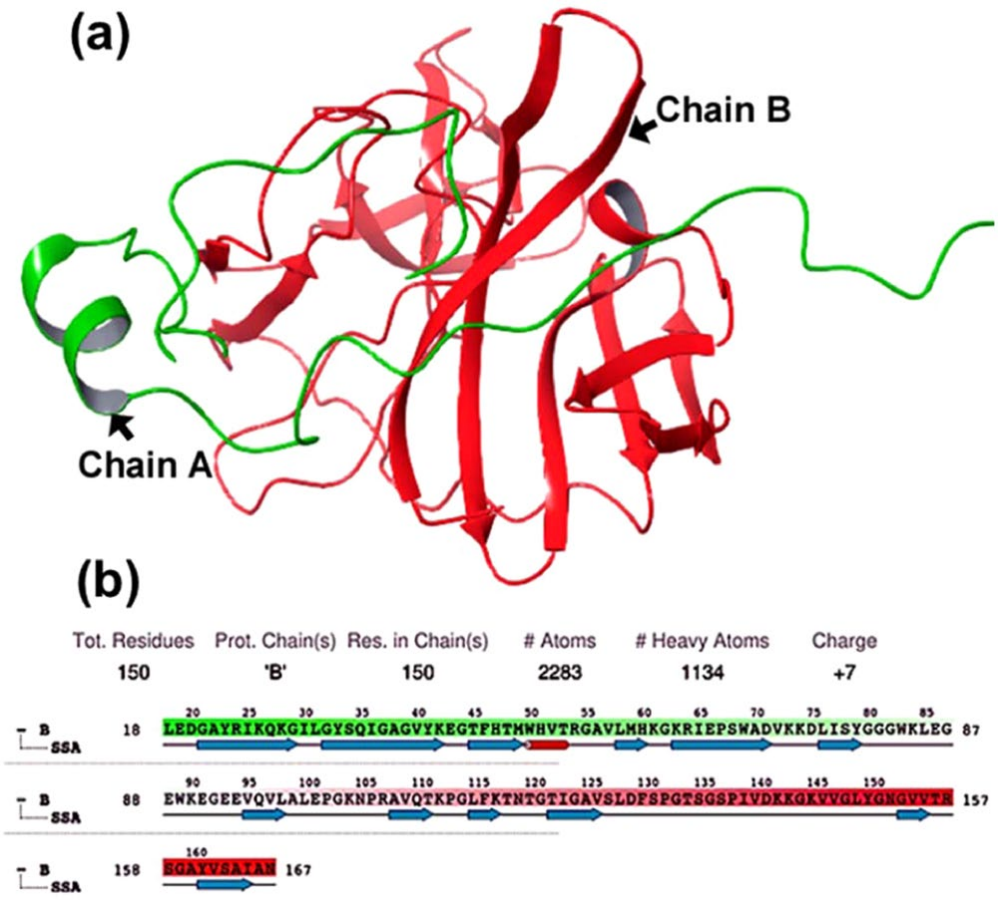
3D structure of DENV NS2B-NS3 protease: (**a**) ribbon diagram of the two protein chains; Chain A (Green color) shows the NS2B cofactor and Chain B (Red color) represents the NS3pro domain, and (**b**) graphical representation of the DENV protease NS3pro domain labelled with the total number of amino acid residues, atoms, heavy atoms and net charge, and predicted secondary structure elements (tube = alpha helix, arrows = beta sheets).

Three-dimensional structure (3D) data of the target protein has been established as a primary requirement for drug discovery. Both X-ray crystallographic structures and homology models generated for target proteins have been used to identify potential ligands from chemical databases, but 3D crystallographic structures have been documented to be more effective than *in silico* generated homology models. Therefore, the 3D structure of DENV NS2B-NS3 protease, which has been proposed as a major therapeutic target against DENV infection, was retrieved from the protein data bank (PDB) with PDB ID:2FOM^40^. The crystal structure of NS2B-NS3pro was resolved at 1.5 Å resolution and exhibited two protein chains, i.e. Chain A folded to form NS2B cofactor and Chain B comprising the NS3pro domain (Fig. 2a). Herein, the protease domain (NS3pro) in Chain B (Fig. 2b) was selected for structure based virtual screening with selected triterpenoids from *G. lucidum*.

### Virtual screening and docking simulation analysis

Recently, structure based virtual screening has gained popularity for discovery of novel lead compounds from large bioactive chemical libraries against selected targets. This approach includes simulated docking of ligands at the active site region of the selected receptor, followed by ranking and scoring of inhibitors^23^. In this work, we conducted structure based virtual screening of 22 known antiviral triterpenoids from *G. lucidum* against NS3pro using the Glide module of the Schrodinger suite (Table S1). These inhibitors were further analysed by the XP docking protocol of the Glide module to gather information on binding energy as well as additional binding patterns with the active site residues. It was observed that all the selected triterpenoids exhibited significant binding affinities towards NS2B-NS3pro except Ganoderic acid G (Table S1). Also, molecular docking of the reference inhibitor 1,8-Dihydroxy-4,5-dinitroanthraquinone against the same active site produced a docking score of −5.377 kcal/mol. Therefore, triterpenoids with docking scores better than a threshold of −5 kcal/mol were selected for molecular interaction analysis. We selected a total of four triterpenoids, i.e. Ganodermanontriol (−6.291 kcal/mol), Lucidumol A (−5.993 kcal/mol), Ganoderic acid C2 (−5.948 kcal/mol) and Ganosporeric acid A (−5.830 kcal/mol) as possible inhibitors for the NS3pro domain of DENV protease. These docking scores were considered significant in comparison to the known inhibitor 1,8-Dihydroxy-4,5-dinitroanthraquinone (−5.377 kcal/mol) and reported bioactive molecules Nimbin (–5.56 kcal/mol), Desacetylnimbin (–5.24 kcal/mol) and Desacetylsalannin (–3.43 kcal/mol) from *Azadirachta indica* with DENV NS3pro^41^. These results indicate the potential of bioactive triterpenoids from *G. lucidum* as inhibitors of DENV protease and suggests that they could be used in the development of anti-viral drugs for DENV infection.

### Molecular interaction and MM/GBSA analysis

**Figure 3:**
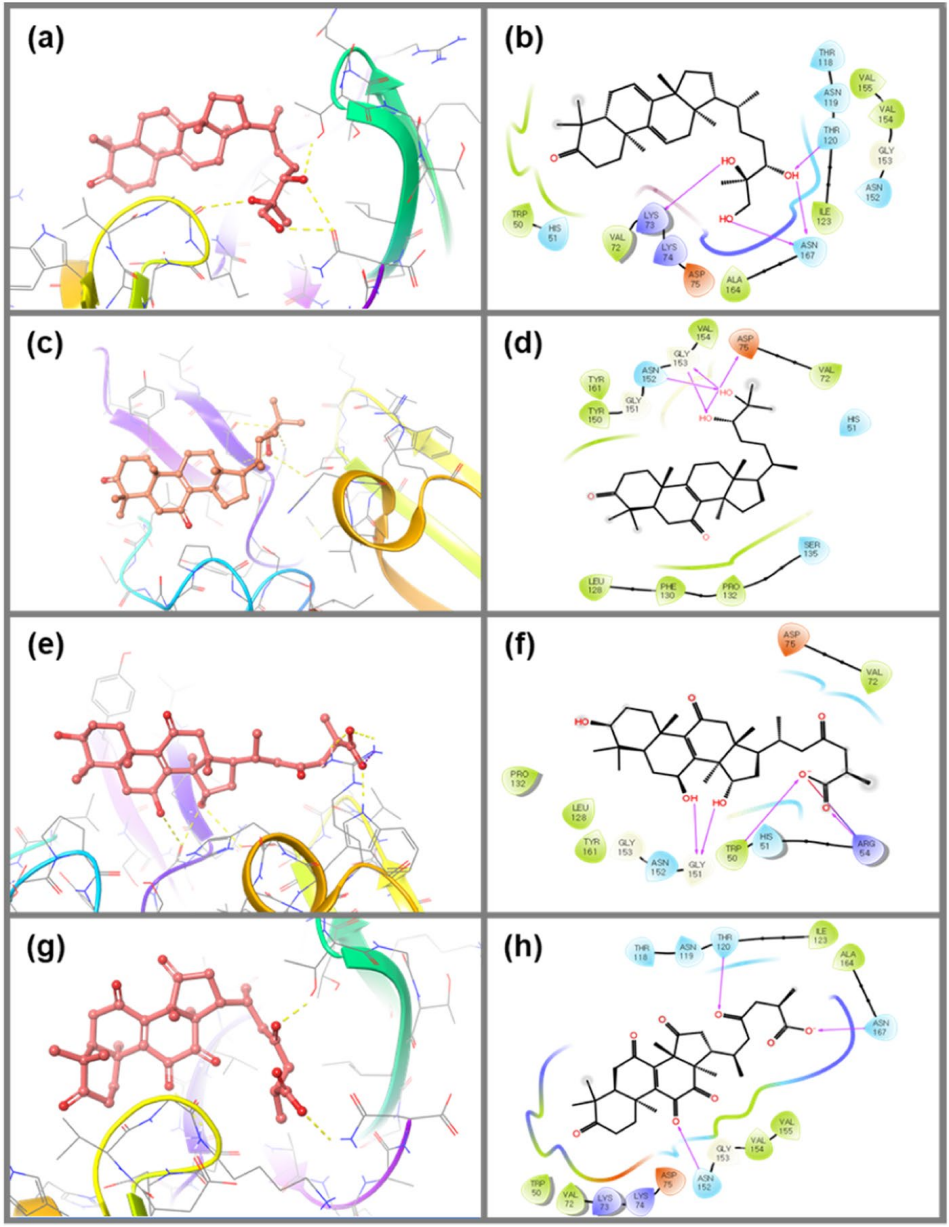
3D and 2D docked complexes of the NS3pro domain exhibiting intermolecular contacts with respective screened triterpenoids; (**a,b**) NS3pro-Ganodermanontriol complex, (**c,d**) NS3pro-Lucidumol A complex, (**e**,**f**) NS3pro-Ganoderic acid C2 (**g**,**h**) NS3pro-Ganosporeric acid A. In 2D complexes, residues in green, violet, red, blue and gray color represents the hydrophobic, positive, negative, polar and glycine interaction, respectively and pink arrows shows the hydrogen bond formation.

Because molecular contacts in protein-ligand docked complexes can lead to improved understanding of molecular mechanisms in biological systems^42^, molecular contact profiling was also studied for the selected triterpenoids and reference inhibitor with DENV protease. The NS3pro-Ganodermanontriol complex exhibited interaction by moderate single and double hydrogen bonds in the active region with Lys73 (3 Å), Thr120 (2.94 Å) and Asn167 (3.26 Å, 2.86 Å) residues, respectively. Additional hydrophobic attractions were also recorded within the NS3pro-Ganodermanontriol complex at residues Trp50, Val72, Ile123, Val154, Val155 and Ala164. Also, residues His51, Thr118, Asn119, Thr120, Asn152, Asn167 and Gly153 exhibited polar and glycine interactions, respectively with Ganodermanontriol. Meanwhile, positive (Lys73 and Lys74) and negative charge interactions (Asp75) with residues were also recorded in the NS3pro-Ganodermanontriol docked complex (Fig. 3a,b).

The interaction profiles of NS3pro-Lucidumol A reflected two single hydrogen bonds formed with residues Asp75 (3.31 Å) and Asn152 (2.48 Å) and double hydrogen bonds formation at Gly153 (2.11 Å, 2.84 Å), which also contributes to a glycine interaction with residue Gly151. Moreover, Hydrophobic (Val72, Leu128, Phe130, Pro132, Tyr150, Val154 and Tyr161) and polar interactions (His51, Ser135 and Asn152) were also logged in the docked complex. Asp75 exhibits negative charge interactions at the active pocket of protease with Lucidumol A (Fig. 3c,d). Also, docked Ganoderic acid C2 complexed with NS3pro displayed moderate double hydrogen bonds formation with Gly151 (2.73 Å, 3.12 Å) while single hydrogen bond formation was observed at residues Trp50 (2.66 Å) and Arg54 (1.93 Å). Moreover, salt bridge formation between the hydroxyl group of Ganoderic acid C2 and Arg54 residue of protease was also recorded in this complex. In addition, polar (His51 and Asn152 residue), negative (Asp75 residue) and positive (Arg54 residue) interactions were also recorded in the NS3pro-Ganoderic acid C2 docked (Fig. 3e,f). The NS3pro-Ganosporeric acid A docked complex showed three single hydrogen bonds formed at residues Thr120, Asn152 and Asn167, while Trp50, Val72, Ile123, Val154, Val155 and Ala164 were marked as exhibiting hydrophobic interactions (Fig. 3g,h). Moreover, the docked NS3pro with Ganosporeric acid A exhibited polar (residues Thr118,Asn119,Thr120, Asn152 and Asn167), positive (Lys73 and Lys74) and negative charge interactions (Asp75).

Additionally, a reference inhibitor, 1,8-Dihydroxy-4,5-dinitroanthraquinone, was also docked at the active site of the NS3pro protease and significant interactions were recorded. This complex exhibits single hydrogen bond formation at residue Phe130, while His51 was noted for salt bridge and pi-pi interaction with the inhibitor. Moreover, hydrophobic (Leu128, Phe130, Pr0132, Tyr150 and Tyr151), polar (His51, Ser131 and Ser135) and glycine (Gly151 and Gly153) interactions were also marked in the docked complex.

It was concluded that all the screened triterpenoids displayed hydrogen bonding with NS3pro at the catalytic triad (His51, Asp75, and Ser135 residue), along with some other conserved residues (Phe130, Tyr150, Asn152 and Gly153) which have been reported to play a significant role in substrate binding for DENV protease^43,44^. Besides, some additional residues were also logged for sharing hydrogen bonds with different ligands. These observations suggest that hydrogen bonds between a ligand and one of the catalytic triad residues of NS3pro can disturb the electron transfer between the carboxyl and imidazole groups of the Asp75 and His51 residues, respectively. Such a disturbance has been reported to lead to an abortive nucleophilic attack of the hydroxyl group (ß-OH) of residue Ser135, which is essential for initiating proteolytic activity^43,44^. Hence, it was concluded that the screened triterpenoids may displayed strong affinity towards the NS3pro domain of DENV through various intermolecular interactions and suggested that they could act as drugs against DENV infection through NS2B-NS3pro inhibition.

**Figure 4:**
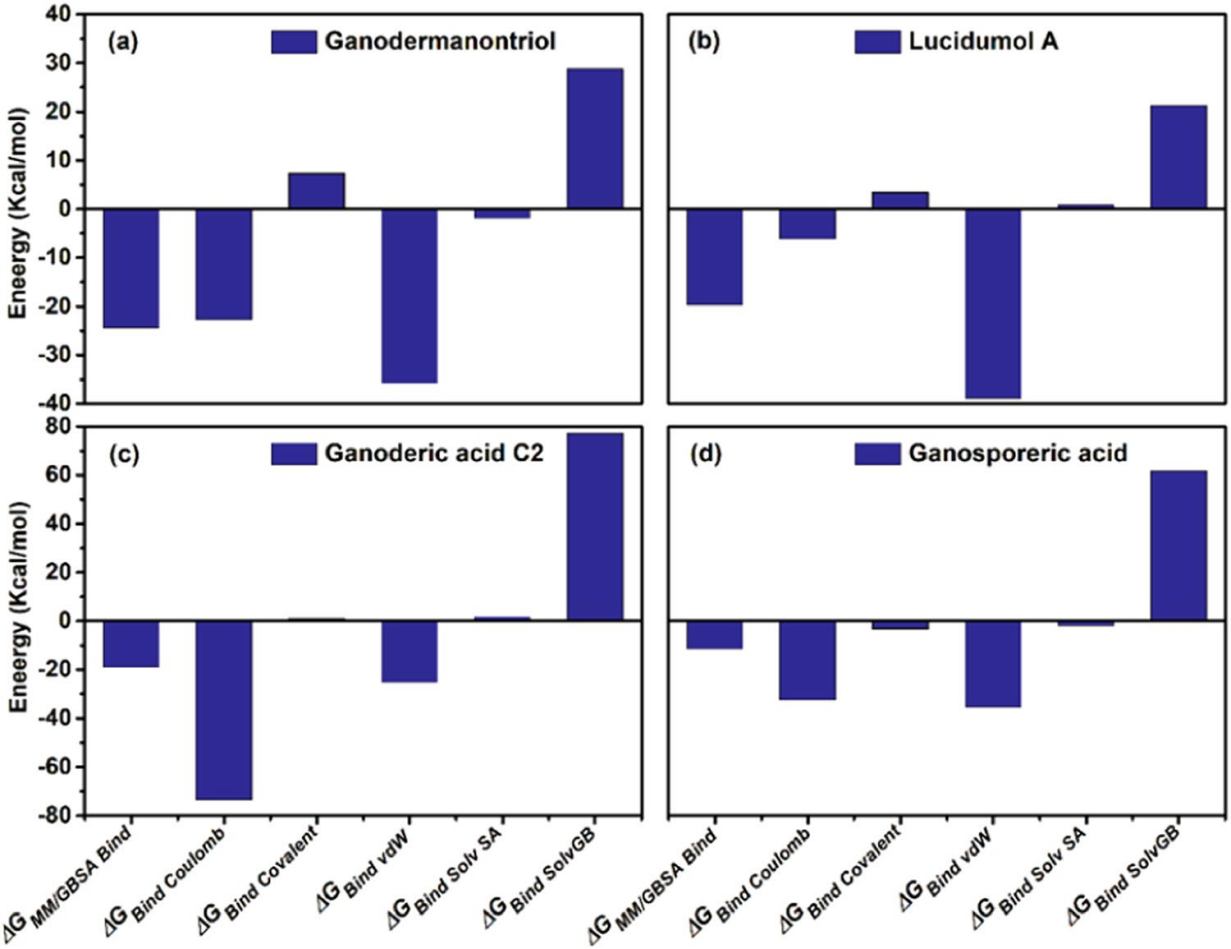
Free binding energy (kcal/mol) calculated using MMGBSA method for screened triterpenoids i.e. (**a**) Ganodermanontriol, (**b**) Lucidumol A, (**c**) Ganoderic acid C2 and (**d**) Ganosporeric acid A complexed with DENV NS2B-NS3 protease after molecular docking simulation.

Furthermore, the docked complexes of all four triterpenoids, Ganodermanontriol, Lucidumol A, Ganoderic acid C2 and Ganosporeric acid A with DENV NS3pro were also analysed using MM/GBSA calculations to calculate the binding affinities of the respective ligands at the active site. These calculations showed relatively negative MM/GBSA values for all four docked complexes, i.e. NS3pro-Ganodermanontriol (−24.465 kcal/mol), NS3pro-Lucidumol A (−19.735 kcal/mol), NS3pro-Ganoderic acid C2 (−19.039 kcal/mol) and NS3pro-Ganosporeric acid A (−11.449 kcal/mol), while the reference inhibitor complex NS3pro-1,8-Dihydroxy-4,5-dinitroanthraquinone produced a larger value (−38.934 kcal/mol). These observations suggest that the inhibition activity of the selected triterpenoids against NS3pro could be weaker than the reference inhibitor, but also suggested the stronger binding affinity of Ganodermanontriol with NS3pro in comparison with the other triterpenoids was supported by predicted docking scores. In addition, predicted *ΔG* values and physicochemical components, that is, *ΔG*_Bind Coulomb_, *ΔG*_Bind covalent_, *ΔG*_Bind vdW_ (van der Waals forces), *ΔG*_Bind Solv SA_ (solvent accessible surface area) and *ΔG*_Bind Solv GB_ (solvation energy generalized Born) analysis for the selected triterpenoids and positive control complexed with DENV NS3pro protein indicates that, depending on the ligand, ΔG_Bind Coulomb_ and/or ΔG_Bind vdW_ contributed the most to the stability of the triterpenoid and reference inhibitor complexes with the DENV NS3pro protein. (Figs. 4, S2, Table S2). Hence, Ganodermanontriol was concluded as the most promising functional triterpenoid for the DENV NS3pro protein, and further analysed along with the other triterpenoids using molecular dynamics and *in vitro* analysis.

### Molecular dynamics (MD) analysis

Interaction information predicted from on target-ligand calculated using the molecular docking approach can be further validated by molecular dynamics simulation and crystallographic studies^38,41^. Herein, the stability of the selected triterpenoid-NS3pro domain complexes was evaluated using 10 ns MD simulation in terms of root mean square deviation (RMSD), root mean square fluctuation (RMSF) and protein-ligand contact map analysis.

**Figure 5:**
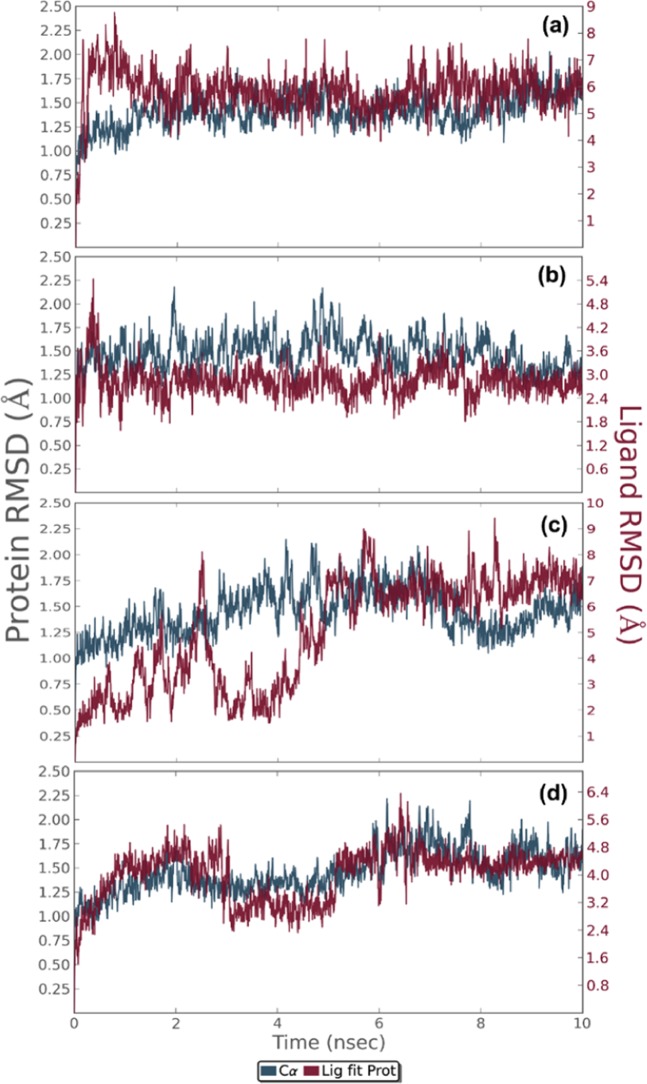
RMSD for alpha carbon atoms of NS3pro protein and triterpenoids from *G. lucidum* as ligand, (**a**) NS3pro-Ganodermanontriol, (**b**) NS3pro-Lucidumol A, (**c**) NS3pro-Ganoderic acid C2 and (**d**) NS3pro-Ganosporeric acid A, in 10 ns trajectory of MD simulations.

The RMSD analysis of C-alpha and backbone atoms in DENV NS3pro complexed with all four ligands showed stable and acceptable deviations during the 10 ns MD simulation (Fig. 5). Interestingly, the final fluctuation for the receptor was recorded as <2 Å RMSD whilst the Ganodermanontriol inhibitor showed a stable and maximum variation of 6 Å in complex with the NS3pro domain at the end of the simulation interval (Fig. 5a). Also, RMSF calculated for both individual residues of the receptor and atoms of the ligand showed tolerable variations (less than 2.8 Å) during the simulation interval (Figs. S3a, S4a). However, insignificant RMSF fluctuations were also observed during a time frame of 5 to 7 ns in the keto and methyl groups of the cyclopenta-phenanthren-7-one ring of the ligand (Fig. S3a).

Likewise, RMSD analysis for the NS3pro-Lucidumol A complex revealed that both receptor and ligand posed variations of less than 3 Å during the 10 ns simulation interval (Fig. 5b). Meanwhile, RMSF analysis for both NS3pro and Lucidumol A predicted variations <3 Å, an acceptable deviations were also recorded in the methyl and hydroxyl group in the heptan region of the ligand during the 10 ns simulation interval (Fig. S3b). Similarly, the ligand in the NS3pro-Ganoderic acid C2 complex showed acceptable deviations during initial 2–5 ns followed by stability and final deviation was recorded at less than 8 Å during the 10 ns simulation interval (Fig. S4b).

The NS3pro-Ganoderic acid C2 complex also exhibited insignificant deviations in the receptor between 2–5 ns followed by stability with final variation recorded at less than 1.75 Å during the simulation interval (Fig. 5c). These acceptable deviations in the ligand (less than 6 Å) and receptor (3 Å) were also supported by the calculated RMSF for the respective complex (Figs. S3c, S4c).

Moreover, simulation analysis of the ligand in the NS3pro-Ganosporeric acid A docked complex exhibited initially low deviation for 6 ns in the carboxylic group located at heptan moiety of ligand initial followed by acquiring a stable state at 4.6 Å RMSD until the end of the 10 ns interval (Fig. 5d). Meanwhile, NS3pro displayed stable oscillations around 1.6 Å RMSD with insignificant deviations in the initial N terminal region (Fig. 5d). These observations suggested the stability of the respective docked complex as supported by the RMSF curves for the individual residues (less than 3.5 Å) and atoms (3 Å) of the receptor and ligand, respectively (Figs. S3d, S4d).

**Figure 6:**
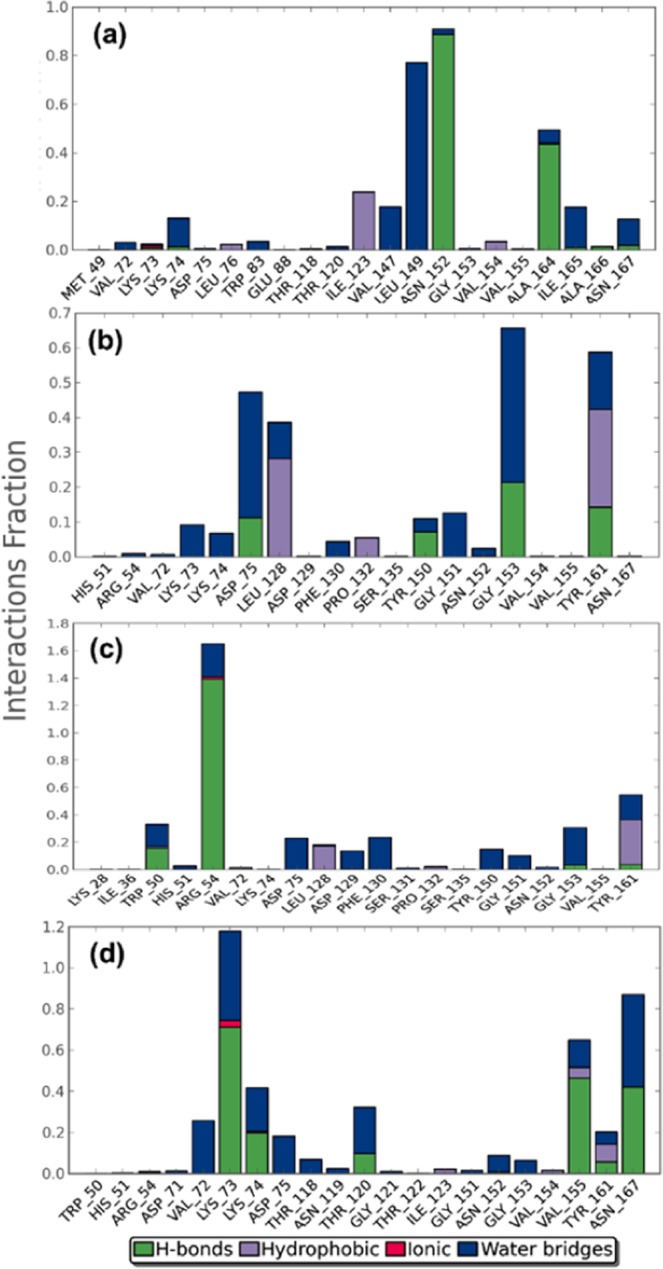
Normalized stacked bar chart for docked complexes, i.e. (a) NS3pro-Gandomanontriol, (b) NS3pro-Lucidumol A, (cc) NS3pro-Ganoderic acid C2 and (d) NS3pro-Ganosporeric acid A with various contacts and interactions profiles calculated during MD simulation. Values of interaction fractions >1.0 are plausible as some residues create multiple interactions of the similar subtype.

A protein-ligand interaction map describing the contribution of individual residues to intermolecular bonding was also generated from the simulation trajectory curves (Fig. 6). The protein-ligand interaction map for the NS3pro-Ganodermanontriol complex displayed participation of Met49, Val72, Lys73, Lys74, Asp75, Leu76, Trp83, Glu88, Thr118, Thr120, Ile123, Val147, Leu149, Asn152, Gly153, Val154, Val155, Ala164, Ile165, Ala166 and Asn167 residues, forming hydrogen bonds, hydrophobic attractions, ionic interactions and water bridges (Fig. 6a). Leu149 and Asn152 residues were highlighted for contributing prominent water bridges and hydrogen bond attractions during the 10 ns simulation interval (Fig. 6a). Residue Asn152 was also predicted to form hydrogen bonds in the docking complex (Fig. 3a,b), suggested the importance of this residue in NS3pro for interaction with Ganodermanontriol.

The NS3pro-Lucidumol A interaction map displayed participation of His51, Arg54, Val72, Lys73, Lys74, Asp75, Leu128, Asp129, Phe130, Pro132, Ser135, Tyr150, Gly151, Asn152, Gly153, Val154, Val155, Tyr161 and Asn167 residues in hydrogen bonds, hydrophobic interactions and water bridging, with Asp75 and Gly153 residues for the largest contributions in water bridging and hydrogen bond formation, respectively (Fig. 6b). Molecular docking analysis also suggested the role of Gly153 in hydrogen bonding with the ligand (Fig. 3c,d), indicated the importance of this residue in protein-ligand stability. Besides, Tyr161, Asp75 and Leu128 residues were also indicated for major contributions in water mediated, hydrogen bond and hydrophobic interactions, respectively, with this ligand (Fig. 6b). These results suggested that Gly153, Tyr161, Asp75 and Leu128 were prominently responsible for the stable conformation of the NS3pro-Lucidumol A complex during simulation interval.

Meanwhile the NS3pro-Ganoderic acid C2 complex exhibited contributions of Lys28, Ile36, Trp50, His51, Arg54, Val72, Lys74, Asp75, Leu128, Asp129, Phe130, Ser131, Pro132, Ser135, Tyr150, Gly151, Asn152, Gly153, Val155 and Tyr161 residues in different molecular interactions in the protein-ligand mapping during the simulation (Fig. 6c). Interestingly, major intermolecular interaction was contributed by Arg54 through hydrogen bond formation followed by water bridges and ionic attraction (Fig. 6c). This residue was also predicted in the molecular docking analysis to form two hydrogen bonds with Ganoderic acid C2 (Fig. 3e,f), suggesting that it is a key factor for maintaining target-ligand stability during the simulation.

Likewise, the NS3pro-Ganosporeric acid A interaction profile showed that Try50, His51, Arg54, Asp71, Val72, Lys73, Lys74, Asp75, Thr118, Asn119, Thr120, Gly121, Thr122, Ile123, Gly151, Asn152, Gly153, Val154, Val155, Tyr161 and Asn167 residues actively contributed to water bridges, hydrophobic and ionic interactions (Fig. 6d). However, Lys73, Lys74, Val155 and Asn167 residues were predicted to make the largest contributions to the stability of NS3pro complexed with Ganosporeric acid A ligand via hydrogen bonds, followed by water bridges, hydrophobic and ionic interactions (Fig. 6d). Also, Asn167 was marked for exhibiting hydrogen bond formation with Ganosporeric acid A in the docked complex, revealing its significant contribution to complex stability. These results validated and suggested the significant stability of each triterpenoid with the NS3pro domain of DENV protease in docked complexes. Hence, it was again suggested that these triterpenoids could be used in the formulation of drugs DENV infection.

## Functional Activity of Triterpenoids

**Figure 7:**
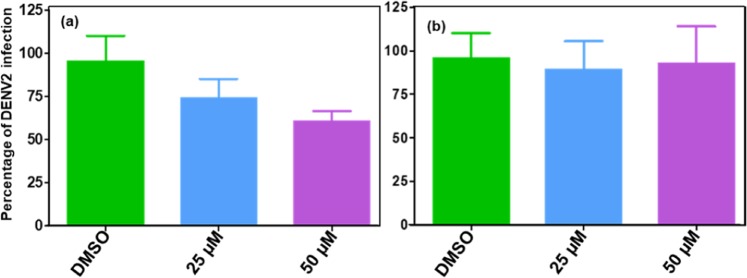
Inhibition activity of DENV2 strain by (**a**) Ganodermanotriol and (**b**) Ganoderic acid C2 was recorded from the reduction in viral titers using plaque assays. Error bars represent geometric means with 95% confidence intervals.

Among the selected triterpenoids, the two compounds i.e. Ganodermanontriol (−6.291 kcal/mol) and Ganoderic acid C2 (−5.948 kcal/mol) were employed in an *in vitro* dengue inhibition assay. It was observed that Ganodermanontriol showed ~40% and ~25% reduction in DENV titers at 50 and 25 µM concentration, respectively (Fig. 7) whilst no significant reduction in viral titers was recorded for Ganoderic acid C2. Also, Ganodermanontriol was previously reported to have activity against Human Immunodeficiency Virus^45^, Epstein-Barr Virus infection^46^ and influenza virus^24^. Thus, Ganodermanotriol is the most promising candidate for the development of a novel drug against dengue virus infection.

In conclusion, based on *in silico* and *in vitro* assessments, Ganodermanotriol was suggested as functional triterpenoid which can be used in the development of natural-product-derived drug for DENV protease against dengue infection.

## Methodology

### Collection of ligands and receptor

The various triterpenoids of *G. lucidum* that have been reported in anti-viral studies^36,45,47^, were searched and retrieved from the PubChem database (https://pubchem.ncbi.nlm.nih.gov)^48^, and considered for screening with DENV protease. Also, the known DENV protease inhibitor 1,8-Dihydroxy-4,5-dinitroanthraquinone was downloaded and used as positive control in the molecular docking analysis^49^. The three-dimensional (3D) structure at fine resolution of 1.5 Å for the viral protein “NS2B-NS3 protease” was collected using PDB ID: 2FOM from the RCSB PDB (http://www.rcsb.org/pdb/home/home.do)^40^. The NS3pro domain in Chain B of the protease was selected for screening with selected triterpenoids. Visualization of the molecular structure of ligand and protein was performed using the Maestro tool of the Schrödinger software modules suite^50^. All other molecular docking and molecular dynamics simulations below were also carried out using Schrödinger software modules.

### Ligand and protein preparation

The selected bioactive triterpenoids were prepared as ligands using the LigPrep module^51,52^ and optimized with the B3LYP/6-31 G** density functional approach^53^. The bond orders in the ligand molecules were adjusted and their various energy minimized conformations were generated in gas phase using OPLS force field^54^. Also, an electron affinity grid map was created with unit van der Waals scaling and partially cut-off at 0.25 value. Moreover, the receptor was kept fixed and ligands were treated as flexible entities during the docking process.

The NS3pro domain of the viral protease enzyme was processed by employing the Prime module^51,52,55^. Co-crystallized water molecules were deleted that could affect ligand interactions with the protein, suitable hydrogen atoms were added according to the hybridization conditions of the carbon atoms and finally protein structure refinement was carried out using the Protein preparation wizard. A conjugate gradient algorithm and distance dependent dielectric constant of 2.0 was employed in subsequent refinement of protein structure to a root mean square deviation of 0.30 Å.

### Active site residues selection and molecular docking

Three residues identified as His51, Asp75 and Ser135^40^, which form the catalytic triad for serine protease activity in DENV NS2B-NS3pro, were selected as the active residues for molecular docking with ligands using the extra precision (XP) docking mode of the Glide 5.8 module^51,55^. The receptor was kept fixed and ligands were treated as flexible entities during the docking studies. Hydrophobic contacts, hydrogen bonds, coulombic, van der Waals, metal binding, and polar interactions, freezing rotatable bonds and a penalty for buried polar groups along with other factors (water desolvation energy and binding affinity enriching interactions) were considered in the Glide XP scoring protocol^53,56^.

### Molecular mechanics/generalized born surface area (MMGBSA) calculations

The free energy calculations for the DENV NS3pro protein docked with selected triterpenoids was conducted using the Prime molecular mechanics/generalized Born surface area (MMGBSA) module as documented previously^57,58^. This approach was applied to the molecular docked simulated complex and free binding energy was then calculated using Equation (1).

1 $$\Delta {G}_{{\rm{MMGBSA}}{\rm{bind}}}=\Delta {G}_{{\rm{complex}}(minimized)}\,-(\Delta {G}_{{\rm{ligand}}(minimized)}+\Delta {G}_{{\rm{complex}}(minimized)})$$

where *ΔG*_MMGBSA bind_ stands for the total free binding energy, *ΔG*_complex_ represents the binding energy of the receptor- ligand complex while *ΔG*_ligand_ and *ΔG*_receptor_ represent the energy for the separated ligand and receptor, respectively.

### Molecular dynamics simulations

Based on the docking scores obtained for different protein-ligand complexes, NS3pro proteins complexed with potential triterpenoid ligands were subjected to molecular dynamics simulations for 10 ns. The designed complexes were fabricated in each direction ( 10Å × 10Å × 10Å buffer) of the gradient box to allow significant conformational fluctuations during MD simulations. Also, TIP4P water molecules were added into the system using minimization of the steepest descent algorithm in 3000 steps trailed by a conjugate gradient algorithm of 5000 steps containing 120 kcal/mol threshold energies. These molecular simulations for protein-ligand complexes were executed in the Desmond v4.4 module of Schrodinger-Maestro v10.4^59,60^. Anisotropic diagonal position scaling on time step interval of 0.002 ps was employed to maintain a constant pressure during MD simulations. Moreover, gradual increment in the system temperature (100 K to 300 K) was allowed along with 20 ps NPT reassembly at 1 atm target pressure. Additionally, the Berendsen algorithm^61^ and Lennard-Jones cut-off value was fixed at 0.2 constant and 9 Å, respectively. Furthermore, SHAKE^62^ ideal limits were imposed on all the chemical bonds including hydrogen atoms. Finally, simulation for each target-ligand complex was performed under the same conditions, system density was maintained near 1 g/cm^3^ and all the calculations were conducted using the OPLS_2005 force field.

### Dengue inhibition assay

For the evaluation of triterpenoids’ functional activity against dengue virus, based on docking score Ganodermanotriol and Ganoderic acid C2 were purchased from ChemFaces Biochemical Co., Ltd. China. The antiviral activity was calculated on A549 cell lines where cells (~40000) were seeded in a 48 well plate and infected with DENV2 strain at 5 multiplicity of infection (MOI) for one hour. Following, the infected cultures were incubated for one hour in the culture media amended with concentrations (25 or 50 µM) of selected triterpenoids. At 24 h post-infection, viral titers in the supernatants were estimated by a plaque assay as described previously^63^.

## Supplementary information

Supplementary Information
