# Supplementary material for: Discovery of Ganoderma lucidum triterpenoids as potential inhibitors against Dengue virus NS2B-NS3 protease
Source: Sci Rep. 2019 Dec 13;9:19059. doi: 10.1038/s41598-019-55723-5 (PMC6911040; doi:10.1038/s41598-019-55723-5)
Supplement: Supplementary file 1 — Supplementary Information [file 41598_2019_55723_MOESM1_ESM.docx]

**Discovery of *Ganoderma lucidum*** **triterpenoids as potential inhibitors against** **Dengue virus NS2B-NS3 protease**

^a^Shiv Bharadwaj^1^, ^b^Kyung Eun Lee^1^, Vivek Dhar Dwivedi ^2^, Umesh Yadava^3^, Aleksha Panwar^4^ Stuart. J. Lucas^5^, Amit Pandey^6^, Sang Gu Kang^1*^

^1^Department of Biotechnology, Institute of Biotechnology, College of Life and Applied Sciences, Yeungnam University, 280 Daehak-Ro, Gyeongsan, Gyeongbuk 38541, Republic of Korea

^2^Centre for Bioinformatics, Computational and Systems Biology, Pathfinder Research and Training Foundation, Greater Noida, India

^3^Department of Physics, Deen Dayal Upadhyay Gorakhpur University, Gorakhpur, India

^4^Clinical and Cellular Virology Lab, Translational Health Science and Technology Institute, NCR-Biotech Science Cluster, Faridabad-Gurgaon Highway, Faridabad-121001. India

^5^ Sabanci University Nanotechnology Research and Application Centre (SUNUM), Istanbul, Turkey

^4^Forest Pathology Division, Forest Research Institute, Dehradun, India

**^a,b^** **Authors contributed equally**

***Corresponding authors**

SGK; Email: [kangsg@ynu.ac.kr](mailto:kangsg@ynu.ac.kr)

**S1. Results and discussion**

**S1.1. Virtual screening and re-docking simulation analysis.**

**Table S1.** Docking results for the selected Triterpenoids from *Ganoderma lucidum* with Dengue virus NS2B-NS3 protease.

| **S.no.** | **Compound** | **Molecular Formula** | **PubChem CID** | **2D structure** | **Binding Score (kcal/mol)** |
| --- | --- | --- | --- | --- | --- |
| 1 | Ganodermanontriol | [C_30_H_48_O_4_](https://pubchem.ncbi.nlm.nih.gov/search/#query=C30H48O4) | 73177 | 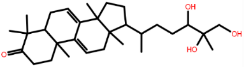 | -6.291 |
| 2 | Lucidumol A | [C_30_H_48_O_4_](https://pubchem.ncbi.nlm.nih.gov/search/#query=C30H48O4) | 475410 | 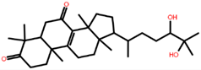 | -5.993 |
| 3 | Ganoderic acid C2 | [C_30_H_46_O_7_](https://pubchem.ncbi.nlm.nih.gov/search/#query=C30H46O7) | 57396771 | 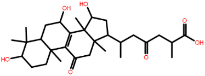 | -5.948 |
| 4 | Ganosporeric Acid A | [C_30_H_38_O_8_](https://pubchem.ncbi.nlm.nih.gov/search/#query=C30H38O8) | 131872 | 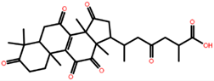 | -5.830 |
| 5 | Ganoderic Acid A | C_30_H_44_O_7_ | 471002 | 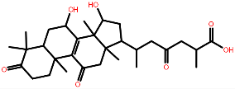 | -5.82 |
| 6 | Lucidumol B | [C_30_H_50_O_3_](https://pubchem.ncbi.nlm.nih.gov/search/#query=C30H50O3) | 475411 | 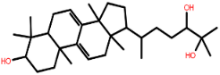 | -5.727 |
| 7 | Ganoderic acid C1 | [C_30_H_42_O_7_](https://pubchem.ncbi.nlm.nih.gov/search/#query=C30H42O7) | 471004 | 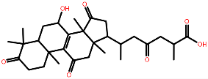 | -5.718 |
| 8 | Ganoderic acid D | [C_30_H_42_O_7_](https://pubchem.ncbi.nlm.nih.gov/search/#query=C30H42O7) | 102004379 | 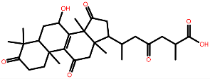 | -5.136 |
| 9 | Ganoderic acid C6 | [C_30_H_42_O_8_](https://pubchem.ncbi.nlm.nih.gov/search/#query=C30H42O8) | 57396921 | 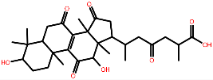 | -5.062 |
| 10 | Ganoderic Acid B | [C_30_H_44_O_7_](https://pubchem.ncbi.nlm.nih.gov/search/#query=C30H44O7) | 471003 | 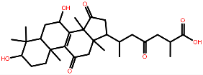 | -5.051 |
| 11 | Ganoderic acid E | [C_30_H_40_O_7_](https://pubchem.ncbi.nlm.nih.gov/search/#query=C30H40O7) | 23247894 | 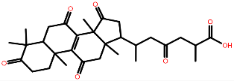 | -5.008 |
| 12 | Ganoderiol F | [C_30_H_46_O_3_](https://pubchem.ncbi.nlm.nih.gov/search/#query=C30H46O3) | 471008 | 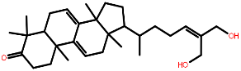 | -4.982 |
| 13 | Methyl Ganoderate A | [C_31_H_46_O_7_](https://pubchem.ncbi.nlm.nih.gov/search/#query=C31H46O7) | 21632954 | 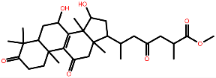 | -4.894 |
| 14 | Ganoderic acid H | [C_32_H_44_O_9_](https://pubchem.ncbi.nlm.nih.gov/search/#query=C32H44O9) | 471005 | 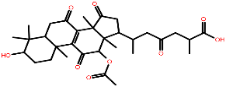 | -4.869 |
| 15 | Ganoderic Acid β | [C_30_H_44_O_5_](https://pubchem.ncbi.nlm.nih.gov/search/#query=C30H44O5) | 6451042 | 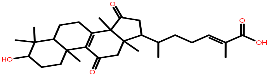 | -4.841 |
| 16 | Methyl Ganoderate B | [C_31_H_46_O_7_](https://pubchem.ncbi.nlm.nih.gov/search/#query=C31H46O7) | 21632955 | 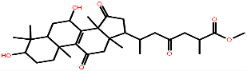 | -4.719 |
| 17 | Ganolucidic Acid D | [C_30_H_44_O_6_](https://pubchem.ncbi.nlm.nih.gov/search/#query=C30H44O6) | 101600076 | 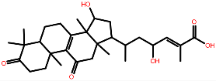 | -4.318 |
| 18 | Ganodermanondiol | [C_30_H_48_O_3_](https://pubchem.ncbi.nlm.nih.gov/search/#query=C30H48O3) | 73294 | 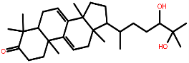 | -4.166 |
| 19 | Ganolucidic Acid A | [C_30_H_44_O_6_](https://pubchem.ncbi.nlm.nih.gov/search/#query=C30H44O6) | 475412 | 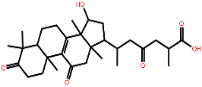 | -4.159 |
| 20 | Ganosporelactone B | [C_30_H_42_O_7_](https://pubchem.ncbi.nlm.nih.gov/search/#query=C30H42O7) | 118705178 | 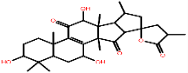 | -3.383 |
| 21 | Ganosporelactone A | [C_30_H_40_O_7_](https://pubchem.ncbi.nlm.nih.gov/search/#query=C30H40O7) | 118705177 | 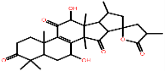 | -1.612 |
| 22 | Ganoderic acid G | [C_30_H_44_O_8_](https://pubchem.ncbi.nlm.nih.gov/search/#query=C30H44O8) | 20055988 | 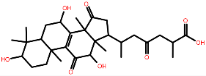 | No binding |

**Molecular Interaction and MMGBSA analysis**

|  |  | **MM-GBSA free energies (kcal/mol)** | | | | |
| --- | --- | --- | --- | --- | --- | --- |
| **S.no** | **Components**  **(kcal/mol)** | **1,8-Dihydroxy-4,5-dinitroanthraquinone** | **Ganodermanontriol** | **Lucidumol A** | **Ganoderic acid C2** | **Ganosporeric acid** |
| 1 | Prime MMGBSA DG bind | -38.934 | -24.465 | -19.735 | -19.039 | -11.449 |
| 2 | Prime MMGBSA DG bind Coulomb | -34.11 | -22.769 | -6.167 | -73.499 | -32.504 |
| 3 | Prime MMGBSA DG bind Covalent | 3.441 | 7.297 | 3.304 | 0.856 | -3.214 |
| 4 | Prime MMGBSA DG bind vdW | -37.819 | -35.834 | -38.925 | -25.235 | -35.411 |
| 5 | Prime MMGBSA DG bind Solv SA | 2.767 | -1.929 | 0.829 | 1.666 | -1.967 |
| 6 | Prime MMGBSA DG bind Solv GB | 26.787 | 28.77 | 21.225 | 77.173 | 61.647 |

**Table S2**. Free binding energy calculations for the selected triterpenoids and psositive control (1,8-Dihydroxy-4,5-dinitroanthraquinone) compounds complexed with DENV NS2pro protein using MMGBSA calculations.

**
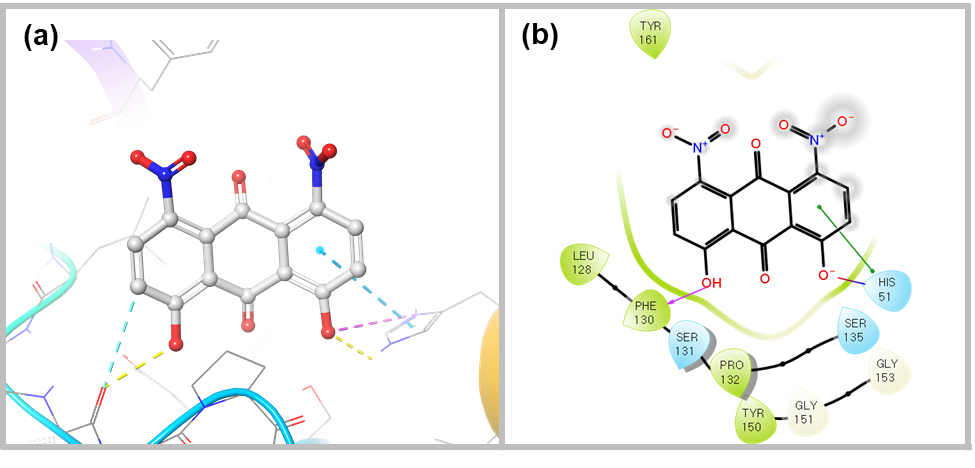
**

**Figure S1.** Molecular docking conformation of 1,8-Dihydroxy-4,5-dinitroanthraquinone in (a) 3D representation and (b) molecular interaction analysis in 2D presentation against the Dengue virus NS2B-NS3 protease. In 2D complexes, residues in green, blue and gray color represents the hydrophobic, polar and glycine interaction, respectively and pink arrows shows the hydrogen bond formation.


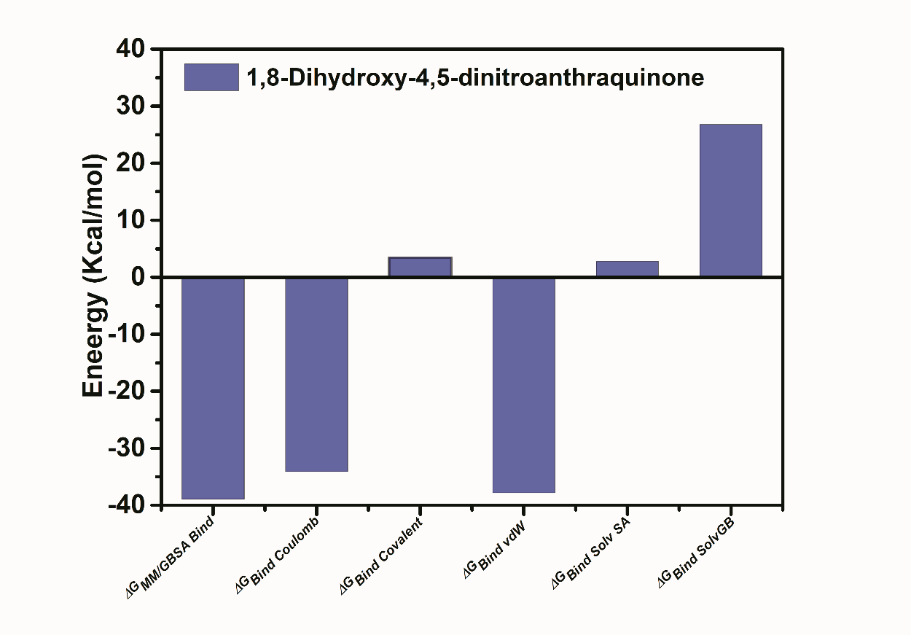


**Figure S2**. Free binding energy (kcal/mol) calculated using MMGBSA method for the positive control (1,8-Dihydroxy-4,5-dinitroanthraquinone) complexed with DENV NS2B-NS3 protease after molecular docking simulation.

**S1.2. Molecular Dynamics Simulation Analysis**


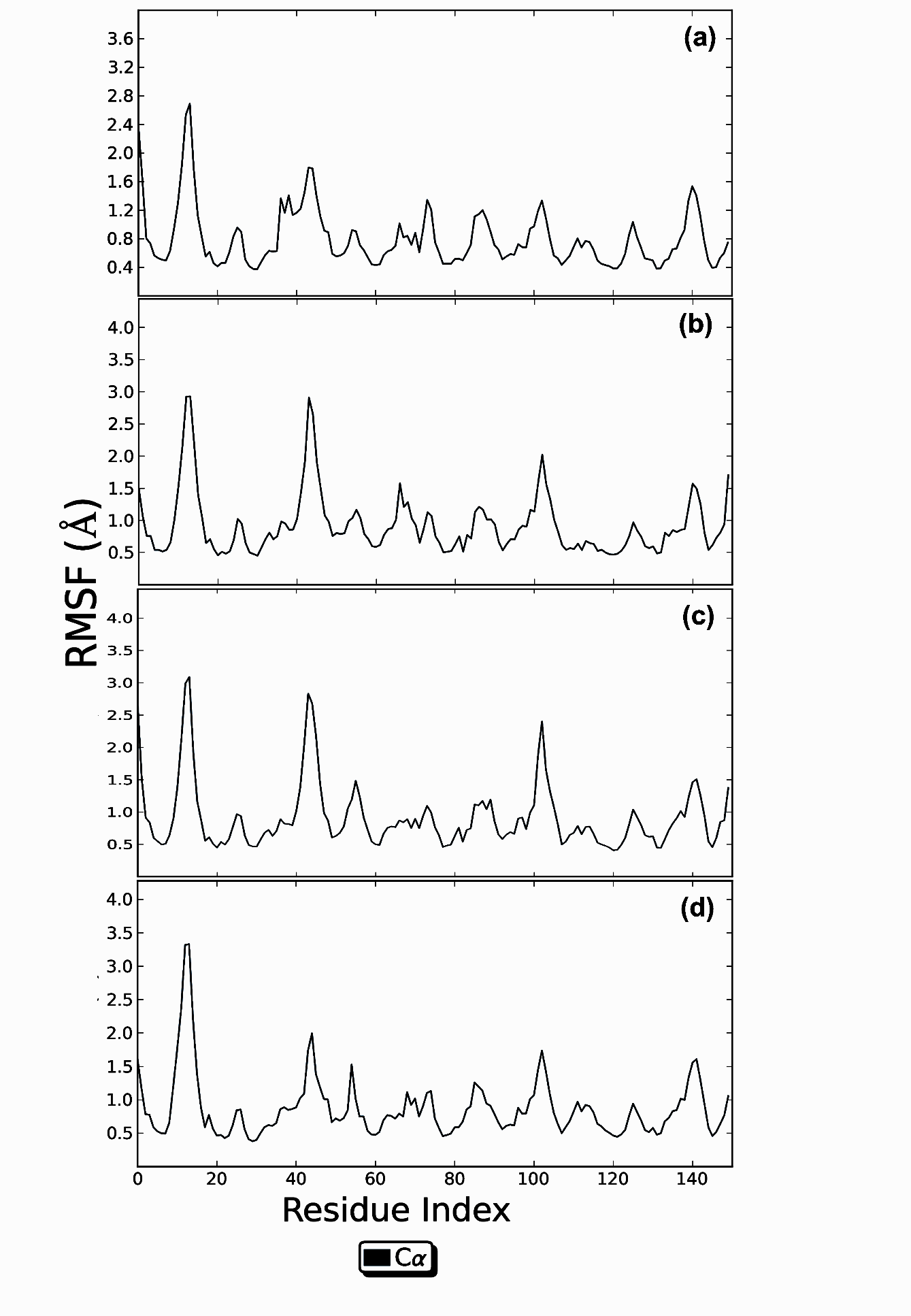


**Figure S3.** Root mean square fluctuation (RMSF) plot of (a) Ganodermanontriol, Lucidumol A, Ganoderic acid C2 and Ganosporeric acid A complexed with DENV NS3pro complex during molecular simulations.


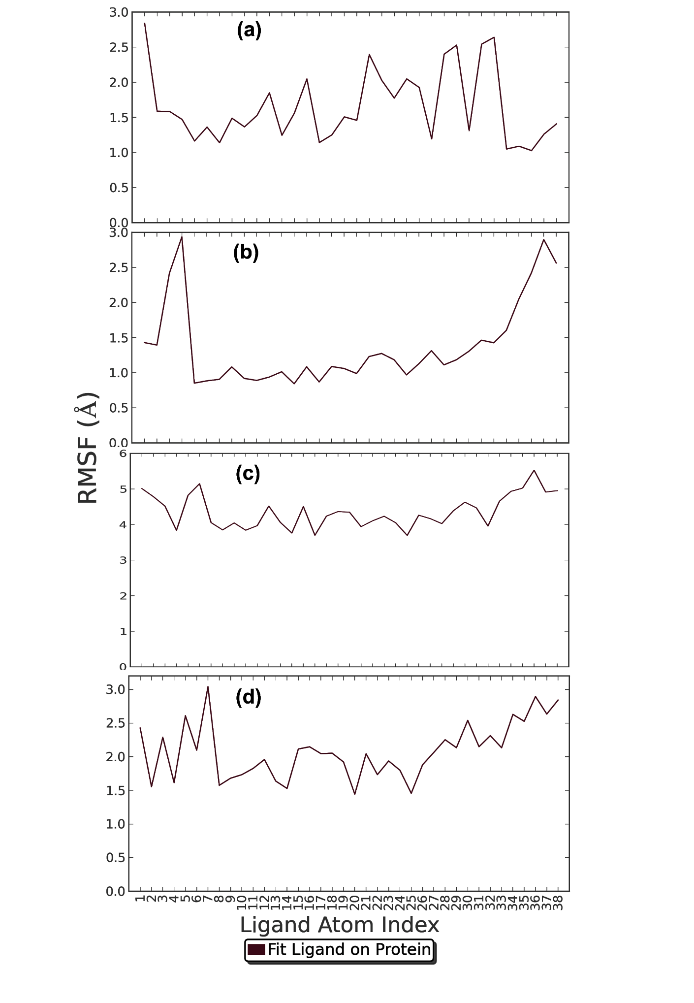


**Figure S4.** Root mean square fluctuation (RMSF) plot of (a) Ganodermanontriol, Lucidumol A, Ganoderic acid C2 and Ganosporeric acid A complexed with DENV NS3pro complex during molecular simulations.
